# Nanog Signaling Mediates Radioresistance in ALDH-Positive Breast Cancer Cells

**DOI:** 10.3390/ijms20051151

**Published:** 2019-03-06

**Authors:** Mozhgan Dehghan Harati, H. Peter Rodemann, Mahmoud Toulany

**Affiliations:** 1 Division of Radiation Biology & Molecular Environmental Research, Department of Radiation Oncology, Eberhard Karls University Tuebingen, 72076 Tübingen, Germany; mozhgan.dehghan-harati@klinikum.uni-tuebingen.de; 2 German Consortium for Translational Cancer Research (DKTK) and German Center for Cancer Research (DKFZ), partner site Tuebingen, 72076 Tübingen, Germany

**Keywords:** ALDH1, Nanog, Akt, Notch, cancer stem cells, radioresistance

## Abstract

Recently, cancer stem cells (CSCs) have been identified as the major cause of both chemotherapy and radiotherapy resistance. Evidence from experimental studies applying both in vitro and in vivo preclinical models suggests that CSCs survive after conventional therapy protocols. Several mechanisms are proposed to be involved in CSC resistance to radiotherapy. Among them, stimulated DNA double-strand break (DSB) repair capacity in association with aldehyde dehydrogenase (ALDH) activity seems to be the most prominent mechanism. However, thus far, the pathway through which ALDH activity stimulates DSB repair is not known. Therefore, in the present study, we investigated the underlying signaling pathway by which ALDH activity stimulates DSB repair and can lead to radioresistance of breast cancer cell lines in vitro. When compared with ALDH-negative cells, ALDH-positive cells presented significantly enhanced cell survival after radiation exposure. This enhanced cell survival was associated with stimulated Nanog, BMI1 and Notch1 protein expression, as well as stimulated Akt activity. By applying overexpression and knockdown approaches, we clearly demonstrated that Nanog expression is associated with enhanced ALDH activity and cellular radioresistance, as well as stimulated DSB repair. Akt and Notch1 targeting abrogated the Nanog-mediated radioresistance and stimulated ALDH activity. Overall, we demonstrate that Nanog signaling induces tumor cell radioresistance and stimulates ALDH activity, most likely through activation of the Notch1 and Akt pathways.

## 1. Introduction

Breast cancer is the most commonly diagnosed cancer in women worldwide. Despite an improvement in the survival rate of patients, breast cancer is still the major cause of death in women aged 20–59 years [1,2]. In general, tumor relapse and metastasis are the prime factors underlying cancer patient death [3]. Several reports have indicated that tumors consist of a heterogenous cell population that includes bulk tumor cells and tumor-initiating/cancer stem cells (CSCs) [4]. CSCs are capable of tumor initiation and self-renewal and can give rise to bulk populations of nontumorigenic cancer cells through differentiation [5]. Moreover, CSCs have been suggested as one of the underlying reasons for tumor recurrence and therapy resistance [3].

CSCs can be isolated from tumor cell subpopulations based on specific surface markers, such as CD44 and CD24, or high aldehyde dehydrogenase1 (ALDH1) enzyme activity [6]. The ALDH family consists of 19 protein isoforms. Among them, the enzyme activity of ALDH1 has been described as a useful prognostic biomarker for poor overall patient survival [7,8,9]. ALDH1 participates in intracellular aldehyde detoxification by catalyzing the oxidation of exogenous and endogenous aldehydes into their corresponding carboxylic acids [10].

Both mesenchymal stem cells, as well as cancer stem cells, have been described to present decreased sensitivity to ionizing radiation [11,12]. In particular, reduced sensitivity of CSCs to radiation therapy (RT) can significantly reduce the efficacy of RT in many cancer patients. Thus, the underlying mechanisms of the altered radiation response of cancer stem cells were and are the subject of many ongoing investigations.

Inhibition of ALDH1 activity has been shown to reduce the resistance of breast CSCs to chemotherapy and radiation therapy. Targeting of ALDH activity in breast CSCs with all-trans retinoic acid (ATRA) or diethylaminobenzaldehyde (DEAB) in combination with doxorubicin or paclitaxel treatment and radiation resulted in significantly reduced cell viability relative to chemotherapy and radiation therapy alone [13]. Moreover, in addition to ALDH1, the importance of other putative CSC markers, especially Nanog, has been discussed and highlighted in the context of different tumor development pathways and stemness features [14,15,16,17]. Nanog protein consists of 305 amino acids and is a key transcription factor, with a role in the self-renewal of undifferentiated embryonic stem cells [18]. In addition, functional studies have demonstrated that Nanog plays a crucial role in tumorigenesis [15,17]. Stimulation and high expression of Nanog in CSC populations from different tumor entities have been shown [19]. Likewise, the induction of Nanog in various tumor cells has been reported to promote drug and radioresistance, as well as upregulation of other CSC markers, such as ALDH1 and CD133 [20,21,22].

Accumulating evidence shows that CSCs confer tumor resistance to radiation therapy via various adaptation mechanisms, such as stimulated DNA-DSB repair capacity and activated PI3K/Akt/mTOR signaling [23,24]. Previous data from our group [24] indicated that a selected radioresistant subpopulation of lung and breast cancer cells presenting the CSC marker ALDH1 can be sensitized to ionizing radiation by treatment with the ALDH1 inhibitor DEAB or the PI3K inhibitor LY294002. In this context, identification of stemness factors involved in radioresistance and investigation of the molecular pathways by which breast CSCs can survive conventional treatment may lead to more efficient cancer therapy. In this study, we show that breast cancer cells in vitro, characterized by high ALDH activity, exhibit Nanog overexpression associated with stimulated postirradiation cell survival. Additionally, inhibition of the Akt and Notch1 signaling pathways abrogated Nanog-mediated ALDH activity and radioresistance of breast cancer cells. Taken together, our data suggest that Nanog signaling is important in triggering the radioresistance of ALDH-positive tumor cells. Thus, strategies targeting Nanog may potentially improve the therapeutic effectiveness of radiation for breast cancer. However, currently, no Nanog antagonist is available, which could be tested for clinical application as well as with respect to side effects in normal tissues.

## 2. Results

### 2.1. ALDH-Positive Cells with Enriched Expression of Putative Stem Cell Markers Show Radioresistance

**Figure 1:**
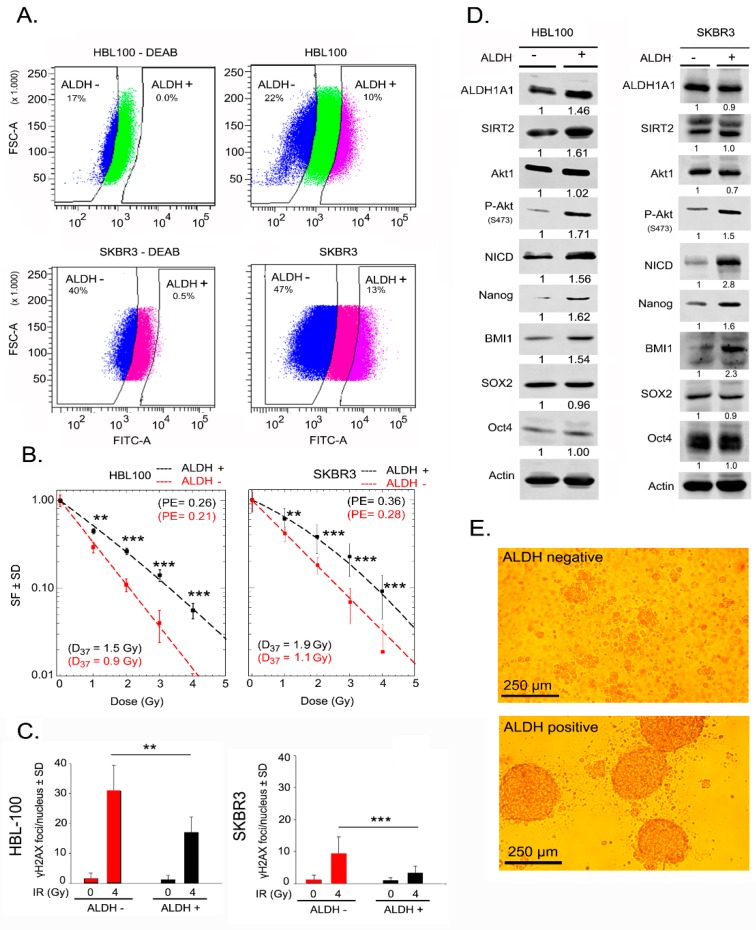
Aldehyde dehydrogenase (ALDH)-positive cells with enriched expression of putative stem cell markers show radioresistance. (**A**) The gating panel of ALDH-positive and ALDH-negative HBL-100 and SKBR3 cells for FACS. Cells were treated with diethylaminobenzaldehyde (DEAB) (40 µM) as a negative control (left panel), and cells without DEAB treatment were the test group (right panel). The gap between ALDH-positive and ALDH-negative cells was induced by prohibiting mixing of the two populations. (**B**) Clonogenic assays of ALDH-positive and ALDH-negative HBL-100 and SKBR3 sorted cells were performed as described in the Materials and Methods Section. Data points represent the mean surviving fractions (SF) ± the standard deviation (SD) from three independent experiments (*n* = 18; ** *p* < 0.01, and *** *p* < 0.001, Student’s t-test, PE = plating efficiency). (**C**) In parallel to the colony formation assay, cells were treated with 4 Gy irradiation, and γ-H2AX foci were analyzed 24 h after irradiation. Survival curves were prepared based on two independent experiments (*n* = 12; ** *p* < 0.01, and *** *p* < 0.001, Student’s t-test). (**D**) Protein expression in ALDH-positive and ALDH-negative HBL-100 and SKBR3 cells. Protein samples were isolated after sorting, and the level of the indicated proteins was analyzed using Western blotting. Densitometry values represent the ratio of the intensity of specific protein bands to that of actin bands normalized to 1 in the DEAB nontreated control cells (ALDH -). (**E**) Sphere formation of ALDH positive and negative HBL-100 sorted cells.

ALDH activity has been described to promote radioresistance in tumor cells from different tissues, such as prostate and breast tissue [23,24]. Thus, to determine whether ALDH-positive breast cancer cells are more radioresistant than ALDH-negative breast cancer cells, both cell populations were isolated from HBL-100 and SKBR3 via FACS (fluorescence-activated cell sorting) based on an Aldefluor assay, see Figure 1A. HBL-100 and SKBR3 cell lines were used due to their higher percentage of basal ALDH activity compared to MCF-7 cells, see Supplementary Figure S1. The gating strategy for the Aldefluor assay and reanalysis of sorted cells is depicted in Supplementary Figure S2. Compared with ALDH-negative cells, ALDH-positive cells from both HBL-100 and SKBR3 cell lines presented a significantly enhanced survival fraction when tested for clonogenic cell survival after exposure to different doses of ionizing radiation, see Figure 1B. Further, to investigate whether radioresistance of ALDH-positive cells is influenced by the efficacy of DNA-DSB repair, we analyzed the residual γ-H2AX foci 24 h post-irradiation. ALDH-positive cells presented a significantly lower number of residual γ-H2AX foci than ALDH-negative cells, see Figure 1C. Moreover, the initial DNA damage (30 min after 1 Gy irradiation) did not show any difference between ALDH-positive and -negative cells, see Supplementary Figure S3. ALDH-positive HBL-100 and SKBR3 cells showed increased expression of the putative stem cell marker proteins Nanog and BMI1 but not Sox2 and Oct4, see Figure 1D. Besides regulating cell proliferation and survival, Notch and PI3K/Akt signaling are important pathways that mediate radiation resistance in tumor cells [25,26]. Therefore, we investigated the role of these pathways in the radioresistance of ALDH-positive sorted cells. In ALDH-positive HBL-100 and SKBR3 cells, phosphorylation of Akt at Ser-473 was strongly increased without a change in the total Akt protein level, see Figure 1D. Likewise, in both cell lines, the expression of Notch intracellular domain (NICD) was greatly increased in ALDH-positive cells, see Figure 1D. Among the different ALDH isoforms tested, the expression level of ALDH1A1 in both ALDH-positive and negative populations was similar, see Figure 1D. In contrast, the expression of the ALDH1A3 isoform is higher in ALDH-positive than in negative cells, see Supplementary Figure S4. In order to determine the role of ALDH activity in CSC phenotype, a sphere formation assay was done for both ALDH positive and negative populations. Compared with ALDH-negative cells, ALDH-positive cells were able to form spheres under a 3D culture condition, see Figure 1E. In addition, we investigated the level of reactive oxygen species (ROS) in ALDH-positive and negative cells. As shown in the Supplementary Figure S5, ALDH-positive cells show a lower level of ROS than ALDH-negative cells. These data indicate that radioresistance mediated by ALDH activity might be partially related to Nanog and Notch1 overexpression and stimulated Akt signaling.

### 2.2. Nanog Expression Correlates with ALDH Activity and Radioresistance

**Figure 2:**
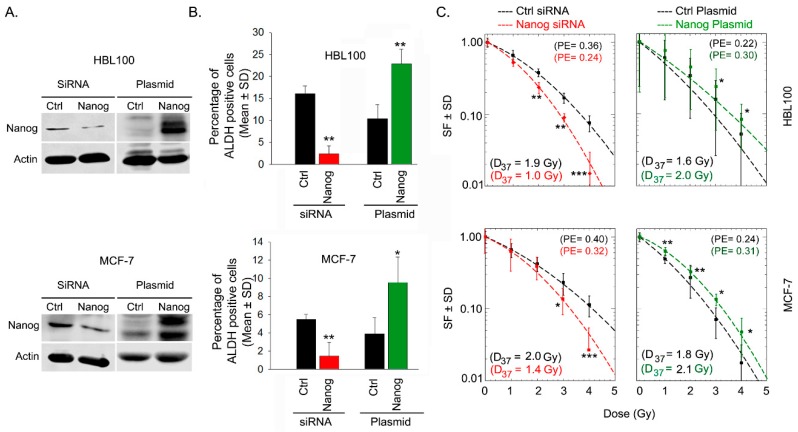
Nanog expression is correlated with ALDH activity and radioresistance. (**A**) Nanog expression was modulated via siRNA and plasmid-based overexpression in the indicated cells as described in the methods. Protein samples were isolated 48 h after cell transfection, and the transfection efficiency was analyzed by Western blotting. (**B**) ALDH activity was measured via an Aldefluor assay using flow cytometry 48 h after transfection. Bars represent the mean relative ALDH activity ± the standard deviation (SD) from three independent experiments (*n* = 6; * *p* < 0.05, and ** *p* < 0.01, Student’s t-test). (**C**) Forty-eight hours after transfection with Nanog siRNA or Nanog expression plasmid, cells were plated for colony formation, irradiated 24 h later and incubated for 10-14 days. Data points represent the mean surviving fraction (SF) ± the standard deviation (SD) from two independent experiments (*n* = 12; * *p* < 0.05, ** *p* < 0.01, and *** *p* < 0.001, Student’s t-test; ctrl: control, PE = plating efficiency).

Based on the previous results, see Figure 1C, we investigated whether Nanog expression affects ALDH activity and, as a consequence, influences the radiation response of HBL-100 and MCF-7 cells. To test this notion, Nanog protein expression was either downregulated by siRNA or induced via transfection with a Nanog expression plasmid, see Figure 2A. The results of Aldefluor assays in both cell lines showed that siRNA-mediated downregulation or overexpression of Nanog led to significant downregulation or upregulation, respectively, of ALDH activity, see Figure 2B, Supplementary Figure S6. Moreover, based on post-irradiation cell survival, siRNA-mediated Nanog downregulation resulted in significant radiosensitization, whereas Nanog overexpression significantly protected both of the tested breast cancer cell lines against radiotherapy, see Figure 2C. These data confirm the importance of Nanog in both ALDH activity and post-irradiation cell survival.

### 2.3. Nanog Expression Stimulates Repair of Radiation-Induced DNA Double-Strand Breaks and Is Associated with Radioresistance of ALDH-Positive Cells

**Figure 3:**
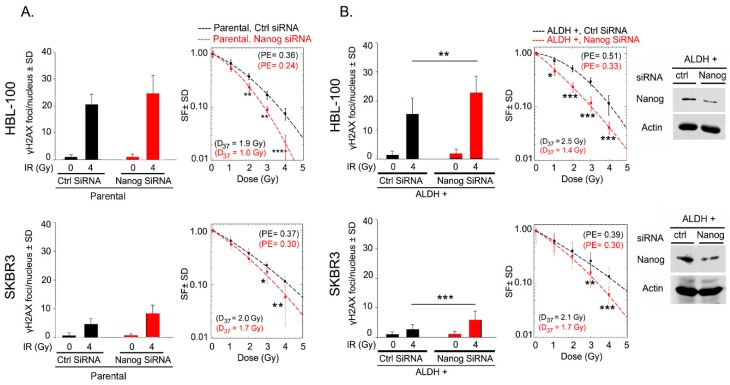
Nanog expression stimulates repair of radiation-induced DNA double-strand breaks and is associated with the radioresistance of ALDH-positive cells. (**A**) and (**B**) Parental and ALDH-positive sorted cells were transfected with 50 nM control (ctrl) or Nanog siRNA and exposed to 4 Gy irradiation 48 h after transfection. Twenty-four hours after irradiation, residual γ-H2AX foci were counted in irradiated and nonirradiated cells. Nanog siRNA-transfected cells were seeded for colony formation assessment and irradiated 24 h later. Survival curves were prepared based on two independent experiments (*n* = 12; * *p* < 0.05, ** *p* < 0.01, and *** *p* < 0.001, Student’s t-test). Protein samples were isolated, and knockdown efficiency was tested by Western blotting.

To investigate whether the stimulated DNA-DSB repair capacity is dependent on Nanog expression, γ-H2AX foci were determined 72 h after Nanog knockdown in parental (not sorted) HBL-100 and SKBR3 cells. siRNA-mediated downregulation of Nanog resulted in a slightly increased number of residual γ-H2AX foci in both cell lines after 4 Gy irradiation, see Figure 3A. Further, to determine the role of Nanog in the DNA-DSB repair capacity of ALDH-positive cells, γ-H2AX foci were determined 72 h after Nanog knockdown in ALDH-positive cells from both cell lines. Downregulation of Nanog resulted in a significantly increased number of residual γ-H2AX foci in ALDH-positive sorted cells from both cell lines, indicating an impaired DNA-DSB repair efficacy in cells with Nanog knockdown, see Figure 3B, Supplementary Figure S7. Moreover, Nanog downregulation significantly reduced the radioresistance of ALDH-positive cells, see Figure 3B. These data indicate that Nanog exerts a regulatory role in the DNA damage response in ALDH-positive cells.

### 2.4. Nanog Promotes ALDH Activity and Radioresistance Through Akt and Notch1 Proteins

**Figure 4:**
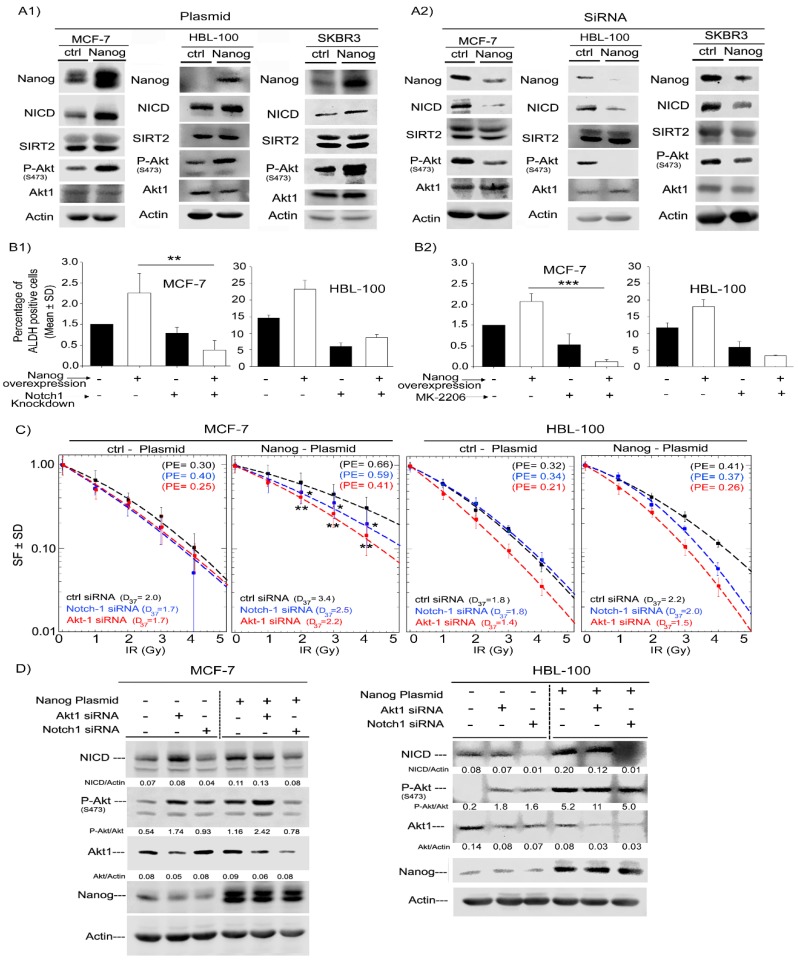
Nanog promotes ALDH activity and radioresistance through Akt and Notch1 proteins. (**A1**) siRNA knockdown of Nanog or (**A2**) Nanog overexpression was performed as described in the Materials and Methods Section in MCF-7, HBL-100 and SKBR3 cells. The transfection efficiency was tested after 48 h by Western blotting. (**B**) ALDH activity as a function of Nanog expression under Notch downregulation and Akt inhibition. Twenty-four hours after Nanog overexpression, cells were transfected with Notch1 siRNA. Forty-eight hours later, ALDH activity was measured with an Aldefluor assay (**B1**). Twenty-four hours after Nanog overexpression, cells were treated with the Akt inhibitor MK-2206 (250 nM for MCF-7 and 1µM for HBL-100), and ALDH activity was measured 24 h later (**B2**). Bars represent relative ALDH activity ± the standard deviation (SD) of three independent experiments (*n* = 6; ** *p* < 0.01, and *** *p* < 0.001, Student’s t-test). (**C**) Survival curve of irradiated cells after Notch1 and Akt knockdown or Akt inhibition in Nanog-overexpressing MCF-7 and HBL-100 cells. Twenty-four hours after Nanog overexpression, cells were transfected with Notch1 siRNA and 24 h later plated for colony formation. Twenty-four hours after plating, cells were irradiated (0 to 4 Gy). Colonies were stained after 10–14 days. MCF-7 data points represent the mean surviving fraction ± the standard deviation of two independent experiments (*n* = 12; * *p* < 0.05, and ** *p* < 0.01, Student’s t-test). HBL-100 data points represent the mean surviving fraction ± the standard deviation of one experiment (*n* = 6) (**D**) Nanog, Akt1 and Notch1 protein expression was analyzed via Western blotting after transfection with plasmid or siRNA under inhibitor treatment conditions.

Next, to determine the mechanism by which Nanog can regulate the radiation response and ALDH activity in breast cancer cells, we assessed the expression and phosphorylation level of Akt and Notch1 in ALDH-positive and ALDH-negative cells. siRNA-mediated downregulation of Nanog in three cell lines led to a decrease in Akt phosphorylation at Ser-473 and a strong decrease in the Notch1 (NICD) protein level. However, the total protein level of Akt and SIRT2, which are downstream of NICD, was not affected, see Figure 4A1. Conversely, overexpression of Nanog increased Akt phosphorylation at Ser-473 and the NICD protein level, see Figure 4A2. Thus, we further asked whether Nanog mediates radioresistance and stimulates ALDH activity through Akt and Notch1 signaling. To address this question, treatment with an Akt inhibitor (MK-2206) and Notch1 knockdown were performed. As shown in Figure 4B1,B2, both MK2206 treatment, as well as Notch1 knockdown, significantly decreased ALDH1 activity in Nanog-overexpressing MCF-7 and HBL-100 cells. Interestingly, only a slight effect was observed in control cells, see Figure 4B. Likewise, knockdown of three different Akt isoforms resulted in a significant decrease in ALDH activity in HBL-100 cells, see Supplementary Figure S8. Furthermore, the same approach was applied to evaluate the cellular radiation response in MCF-7 and HBL-100 cells. siRNA-mediated knockdown of Akt1 and Notch1 abrogated the radioprotective effect of Nanog. However, this treatment did not strongly affect the radiation response of control cells, see Figure 4C. Moreover, the radiosensitizing effect of MK-2206 treatment was less effective than that of the Akt1 siRNA approach, see Supplementary Figure S9. The applied concentration of MK-2206 in this study has been defined based on the preliminary dose kinetic experiment for each cell line and the lowest concentration in which Akt activity is completely blocked has been chosen. As confirmed in Figure 4D, knockdown of Akt1 and Notch1 in both cell lines clearly affected Notch1 and Akt protein expression and phosphorylation of Akt at Ser-473 based on immunoblotting analysis. Thus, the data presented in Figure 4 indicate that Nanog stimulates ALDH1 activity and mediates the radioresistance of tumor cells in part through the Akt and Notch pathways.

## 3. Discussion

High ALDH1 activity is well recognized to serve as a CSC marker in different tumor types [9,27,28,29]. ALDH1 activity plays an important role in several prominent biological activities which lead to the maintenance and progression of different cancer types [30,31,32,33]. However, the relationship between ALDH1 activity and radiation response is largely unknown. Our previous report and reports from other studies demonstrated that ALDH1-positive sorted cells show a better radiation response than ALDH-negative sorted cells [23,24,34], but to date, the underlying mechanism of ALDH1-mediated radioresistance remains unclear and requires further investigation.

In the current study, we showed that the protein expression level of putative stem cell markers, such as Nanog and BMI1, is increased in ALDH-positive sorted cells compared with ALDH-negative cells. This finding is in agreement with other reports [35,36,37]. For example, Yu et al. [35] reported that the mRNA levels of BMI1 and Snail were significantly elevated in ALDH-positive head and neck squamous cell carcinoma (HNSCC) cells. Additionally, Zhi et al. [36] observed higher Nanog mRNA levels in the gastric cancer cell line SNU-1, which presents increased ALDH mRNA levels. Furthermore, compared with ALDH-negative sorted cells, ALDH-positive cells showed increased phosphorylation of Akt at Ser-473 and increased expression of NICD. In line with these data, several recent studies have reported that Notch1 and PI3K/Akt signaling are strongly correlated with ALDH activity [38,39,40,41].

The topic of DNA damage repair activity in CSCs has been addressed by various studies [42,43,44,45]. In this regard, Bao et al. [42] reported that CD133-positive glioblastoma CSCs show stronger ATM and Chk1 protein activation than CD133-negative cells. As a consequence, CD133+ cells survive better when exposed to ionizing radiation [42]. Moreover, it has been observed that the DNA copy number of BRCA1 and RAD51, both of which are involved in DNA-DSB repair via the homologous recombination pathway, is significantly increased in prostatic CSCs [45].

In the present study, we demonstrated that ALDH-positive compared with ALDH-negative sorted cells exhibit significantly improved capacity for repair of DNA-DSBs. These data are in agreement with results reported by Cojoc et al. [23] that indicated that ALDH-positive prostate cancer cells present an enhanced DNA repair capacity, as well as highly activated ATM–Chk2 signaling. Furthermore, Meng et al. [46] reported that stable knockdown of ALDH1A1 in the platinum-resistant ovarian cell line A2780/CP70 leads to a strong increase in BRCA1 and γ-H2AX protein expression, which indicates impaired DNA-DSB repair ability in the absence of ALDH1A1. In addition, we showed that ALDH-positive cells are able to form spheres under 3D culture condition and they present lower levels of ROS than ALDH-negative cells. In line with presented data, there are several studies reporting that CSCs have lower intracellular ROS levels associated with an increased expression of free radical scavengers [47,48]. Further, we observed that the initial DNA damage in both ALDH-positive and -negative populations is the same. These data confirm that lower levels of ROS do not affect the initial repair capacity of ALDH-positive cells.

Nanog upregulation has been reported to be correlated with self-renewal, immune evasion, and resistance to drug and radiation treatment. These are features that reflect CSC characteristics [16,17,21,22]. In this context, Jeter et al. [21] observed increased resistance of Nanog-overexpressing MCF-7 cells to chemotherapeutic drugs, such as doxorubicin and paclitaxel. In addition, these Nanog-overexpressing cells exhibited stimulated ALDH activity, as well as increased expression of the CSC marker CD133 [21]. Likewise, in the present study, we showed that Nanog downregulation or overexpression resulted in decreased or upregulated ALDH activity, respectively. Additionally, we demonstrated that the radioresistance and DNA repair capacity of ALDH-positive sorted cells was dependent, in part, on Nanog expression. In line with these findings, Tanno et al. [22] provided evidence that the stimulated clonogenic potential of medulloblastoma stem-like cells exposed to ionizing radiation is strongly dependent on the upregulation of Nanog protein expression. Likewise, in their study, upregulation of Nanog at the mRNA level was observed from 24 to 72 h post irradiation [22].

Consistent with our results showing strong coexpression of Nanog, Notch and P-Akt in ALDH-positive sorted cells, we identified an interaction between Nanog and the Notch and PI3K/Akt pathways that has not been previously described. This association was evidenced by the results indicating that knockdown of Notch1 and Akt1 abrogates Nanog-mediated radioresistance and Nanog-mediated high ALDH activity in breast cancer cell lines. With respect to the role of the PI3K/Akt pathway in the DNA repair ability of CSCs, it has been reported that pharmacological inhibition of Akt impairs DNA-DSB repair in mouse mammary CSCs via inhibition of Wnt/beta-Catenin signaling [49]. Moreover, previous work from our laboratory demonstrated that targeting of the PI3K/Akt pathway, as well as the selective inhibition of PI3K or Akt, impairs the efficacy of DNA-DSB repair, primarily through the DNA-PKcs-dependent nonhomologous end-joining mechanism [25,50]. As a consequence, the survival of tumor cells exposed to ionizing radiation is significantly impaired [50]. In the present study, we showed that knockdown of Akt1 reduced the radioresistance of Nanog-overexpressing MCF-7 cells. However, in these cells, we were not able to detect a similar strong radiosensitization when Akt activity was inhibited by MK-2206. This result might be due to additional Nanog-mediated pathways involved in the cellular radiation response, which need to be further investigated. For example, Lin et al. [51] reported that p53 negatively regulates Nanog via binding to the Nanog promoter and thus suppresses Nanog transcription in response to DNA damage. A lack of p53 activation after ionizing radiation has also been suggested to be correlated with Nanog overexpression [22]. Moreover, it has been shown that complexation of Nanog and phosphorylated STAT3 leads to expression of microRNA-21, which results in chemoresistance in HNSCC cells [52]. In a recent study, the role of BMX-STAT3 in glioblastoma radioresistance was demonstrated, indicating that pharmacological inhibition of BMX-mediated STAT3 activation in combination with radiotherapy effectively suppresses tumor growth in glioblastoma-bearing mice [53]. These results may indicate that additional mechanisms exist through which Nanog may play a role in DNA repair and cellular radiation response.

Whether ALDH-positive sorted cells indeed resemble a pure CSC population is not clear because there are various CSC markers, such as CD44 +/CD24 –, which are also known as indicators of CSCs in breast cancer [54]. In this context, Liu et al. [55] reported that CSC markers are associated with various subpopulations rather than identifying a common subpopulation in tumor tissues. In this study, it was also found that the CD44+ and CD24- phenotype is equally distributed among ALDH-positive and ALDH-negative MDA-MB-468 breast cancer cells [55].

Herein, we report for the first time that Nanog regulates ALDH activity and the cellular radiation response through the Notch1/Akt signaling pathway. However, the key role of Nanog as a transcription factor that regulates several aspects of cancer development is widely accepted [19]. In the current study, we demonstrated a significant and important role of Nanog in the radiation response of putative breast cancer stem cells. Thus, the function of Nanog as a transcription factor for genes involved in DNA-DSB repair needs to be further investigated. Our data suggest that Nanog signaling is an important component of the radiation response of ALDH-positive putative CSCs, which induce radioresistance through activation of the Notch1 and Akt pathways. Consequently, developing strategies to target Nanog may help improve the therapeutic effectiveness of radiation for breast cancer treatment.

## 4. Materials and Methods

### 4.1. Cell lines, Antibodies and Reagents

The MCF-7 (ATCC^®^ HTB-22™), SKBR3 (ATCC^®^ HTB-30™) and HBL-100 (ATCC^®^ HTB-124™) breast cancer cell lines were used in this study. Cells were cultured in RPMI-1640 (Invitrogen, Gibco) supplemented with 10% fetal bovine serum (FBS) and 1% penicillin-streptomycin. An Aldefluor^®^ assay kit was purchased from Stem Cell Technologies (Vancouver, Canada). Antibodies against P-Akt (Ser-473) (cat. # 4060), Akt1 (cat. # 2967), Nanog (cat. # 3580), Sirt2 (cat. # 12672), Notch1 (cat. # 3608), BMI1 (cat. # 5856), Oct-4 (cat. #7 5463), Sox2 (cat. # 4900), and ALDH1 (cat. # 36671) were purchased from Cell Signaling (Frankfurt, Germany). Anti-Actin antibody (cat. # A2066) was purchased from Sigma (Taufkirchen, Germany). Anti-phospho-Histone H2AX (Ser139) antibody (cat. # 05-636) was purchased from Merck Millipore (Darmstadt, Germany). The Akt inhibitor MK2206 (cat. # S1078) was purchased from Selleckchem (Houston, TX, USA). Gamma-Secretase Inhibitor IX (GSI) (cat. # 565770) was purchased from Calbiochem. siRNA against Nanog1 (cat. # M-014489-02), Akt1 (cat. # M-003000-03), and Notch1 (cat. # M-007771-02) and nontargeting siRNA (cat. # D-001810) were obtained from Dharmacon (Lafayette, IN, USA), and the pCDNA3.1-Nanog plasmid were purchased from Addgene (Cambridge, MA, USA).

### 4.2. Aldefluor Assay and Fluorescence-Activated Cell Sorting

The Aldefluor^®^ assay was performed according to the manufacturer’s instructions. Cell suspensions were incubated with activated ALDEFLUOR reagent and BODIPY-aminoacetaldehyde (BAAA) at 37 °C for 40 min. As a negative control, cells were additionally treated with DEAB (40 µM) under the same conditions. Cells were sorted and analyzed via fluorescence-activated cell sorting (FACS) using a FACSCanto II system and FACS ARIA software, respectively (BD Biosciences, Bergen, NJ, USA). Dead cells were excluded by using 1 µg/mL propidium iodide antibody. A gap between the ALDH-positive and ALDH-negative gates was made to avoid mixing of these two populations during sorting. For comparison of ALDH activity under different test conditions, the percentage of activity under control conditions was normalized to 1.0.

### 4.3. Cell Transfection with siRNA or Plasmid and Immunoblotting

Cells were transiently transfected with 50 nM siRNA against Nanog, Akt1, or Notch1 or with nontargeting siRNA using Lipofectamine based on the manufacturer’s instructions. Immunoblot analysis was performed two days after transfection. To extract protein for immunoblotting, cells were washed with PBS (phosphate buffered saline), and cell lysis was performed with lysis buffer containing phosphatase and protease inhibitors. Protein extracts were finally prepared after short sonication and centrifugation (13,580× *g*, 15 min, 4 °C) procedures.

### 4.4. Colony Formation Assay and γ-H2AX Foci Analysis

Postirradiation cell survival was analyzed using a standard colony formation assay. Cells were preplated in 6-well plates and, 24 h after plating, mock irradiated or irradiated with the doses indicated in each experiment. Irradiation was performed using a Gulmay RS225 X-ray machine (Gulmay Ltd., 293 Chertsey, UK) operating at 200 kVp, 15 mA and with an additional 0.5-mm copper filter. After incubation for 10–14 days, the cells were fixed and stained with crystal violet, and colons with more than 50 cells were counted. The radiation dose required 37% cell survival, which is defined as D_37_ value, was calculated for each dose–response curve by SigmaPlot software.

Plating efficiency (PE) is one important parameter in radiation biology, which allows the evaluation of radiation effects on cellular growth and survival clonogenicity, i.e., cell survival. Survival fraction is calculated based on PE of irradiated cells divided by the PE of non-irradiated control cells. PE is calculated based on the number of colonies with more than 50 cells divided by the number of cells seeded.

γ-H2AX foci were used to evaluate the repair of irradiation-induced DSBs as previously described [50]. To this end, cells were grown on chamber slides and exposed to 4 Gy irradiation. After 24 h, the cells were fixed, permeabilized and blocked with 70% ethanol, 0.2% Triton X-100 and 3% BSA (bovine serum albumin), respectively. After blocking, the fixed cells were incubated with anti-phospho-histone H2AX (Ser-139) antibody, followed by incubation with the secondary antibody. The residual γ-H2AX foci were imaged using a fluorescence microscope (Axioplan 2, Zeiss, Jena, Germany). The average number of residual γ-H2AX foci per cell 24 h after irradiation was calculated on the basis of at least 150 nuclei analyzed in three independent experiments.

### 4.5. Sphere Formation Assay

Spheroid formation of ALDH-positive and -negative sorted cells were determined by culturing 6000 cells with 200 µL medium into 1.5% agarose-coated 96-wells plates. Sphere formation was monitored by phase-contrast microscopy and spheres were counted 4 days after seeding [56].

### 4.6. ROS Detection Assay

A reactive oxygen species (ROS) measurement was performed according to the manufacturer’s instructions (Abcam, Cambridge, UK). ALDH-positive and negative sorted cells (2 × 10^5^ cells) were incubated with oxidative stress detection reagent (2.5 µM) at 37 °C for 30 min. As a negative control, cells were additionally treated with ROS inhibitor (N-acetyl-Lcysteine) under the same conditions. The data analyzed via fluorescence-activated cell sorting (FACS) using a FACSCanto II system equipped with a blue laser (488 nm filter).

## Supplementary Materials

Supplementary materials can be found at https://www.mdpi.com/1422-0067/20/5/1151/s1.

## Abbreviations

CSCs	Cancer stem cells
DSB	DNA double-strand break
ALDH	Aldehyde dehydrogenase
NICD	Notch intracellular domain
DEAB	Diethylaminobenzaldehyde
ATRA	All-trans retinoic acid
