# Supplementary material for: Nanog Signaling Mediates Radioresistance in ALDH-Positive Breast Cancer Cells
Source: Int J Mol Sci. 2019 Mar 6;20(5):1151. doi: 10.3390/ijms20051151 (PMC6429380; doi:10.3390/ijms20051151)
Supplement: Supplementary file 1 [file ijms-20-01151-s001.zip › Figure legends of Supplementary data.pdf]

Supplementary data figure legends

Figure S1. ALDH activity in different breast cancer cells. (A) HBL-100, SKBR3 and MCF-7 were treated with DEAB (40 μM) as a negative control and the gate for positive activity was determined based on their negative sample. HBL-100 and SKBR3 show higher ALDH activity compare to MCF-7. **(B)** The average percentage of ALDH positive cells in the three cell lines has been calculated from three independent experiments (n=3).

Figure S2. Gating and sorting strategy based on ALDH activity. HBL-100 cells were treated with DEAB (40 μM) as a negative control, and the gate for high and low activity was determined based on the negative sample **(A)**. After 3 h, among the entire sorted cell population, 18% showed high activity **(B)**. Reanalysis of the negative **(C)** and positive **(D)** sorted cells showed the purity and accuracy of sorting. After sorting, the cells were directly cultured in RPMI medium with 10% FBS for further experiments.

Figure S3. Initial DNA-DSB repair in ALDH-positive and ALDH-negative cells. ALDH-positive and ALDH-negative HBL-100 and SKBR3 sorted cells were irradiated 1 Gy and γ-H2AX foci were analyzed 30 min after irradiation. Residual γ-H2AX foci were counted in irradiated and non-irradiated cells.

Figure S4. ALDH1A3 expression in ALDH-positive and ALDH-negative cells.

Protein expression in ALDH-positive and ALDH-negative HBL-100 as well as SKBR3 cells were analyzed by Western blotting. Densitometry values represent the ratio of the intensity of specific protein bands to that of actin bands.

Figure S5. ROS level in ALDH-positive and ALDH-negative cells.

ALDH-positive and negative SKBR3 and HBL-100 cells (2×10^5 cells) were incubated with oxidative stress detection reagent (2.5 μM) at 37 °C for 30 min as described in Method section. Upon incubation, the fluorescent products generated by the detection reagent was visualized via fluorescence-activated cell sorting (FACS) using a FACS Canto II system.

Figure S6. ALDH activity based on Nanog expression in MCF-7 cells. The ALDH activity in MCF-7 cells was measured using an Aldefluor assay kit 48 h after transfection with 50 nM nontargeting siRNA (control) or Nanog siRNA **(A)** or 650 ng/µl backbone plasmid (control) or Nanog overexpression plasmid **(B)**. The negative sample represents cells treated with DEAB (40 µM).

Figure S7. γ-H2AX foci in ALDH-positive and ALDH-negative sorted cells. ALDH-positive HBL100 and SKBR3 sorted cells were transfected with 50 nM control (ctrl) or Nanog siRNA. Then, 48 h after transfection, cells were exposed to 4 Gy irradiation, and 24 h later, γ-H2AX foci were analyzed with a fluorescence microscope. Magnification for all figures is 400X.

Figure S8. Effect of Akt isoforms on ALDH activity in HBL100 cells. (A1) The ALDH activity in HBL-100 cells 48 h after transfection with 50 nM nontargeting siRNA (control) or siRNA targeting different Akt isoforms was investigated with an Aldefluor assay. Bars represent the mean surviving fraction ± the standard deviation of three independent experiments. Error bars, SD.(**P<0.01, and ***P<0.001, Student's t-test). **(A2)** Protein samples were isolated, and the effect of knockdown of the three Akt isoforms was tested via immunoblotting**. (B1)** ALDH activity was measured via an Aldefluor assay using FACS, 48 hours after Akt-1 overexpression (650 ng/µl) in HBL-100 cells. **(B2)** The gating for ALDH activity after overexpression of Akt1.

Figure S9. Nanog promotes radioresistance through Akt protein. (A) Survival curve of irradiated cells after 2 h treatment with MK-2206 (250 nM in MCF-7 cells and 1 µm in HBL-100 cells) in Nanog-overexpressing MCF-7 and HBL-100 cells. Forty-eight hours after Nanog overexpression, cells were plated for colony formation. Twenty-four hours after plating, cells were treated with MK-2206 followed by irradiation 2 h after treatment. Colonies were stained after 10-14 days. (B) Protein samples were isolated, and overexpression efficiency and inhibition of Akt was tested by Western blotting.
