# Supplementary figures and images for: Nanog Signaling Mediates Radioresistance in ALDH-Positive Breast Cancer Cells
Source: Int J Mol Sci. 2019 Mar 6;20(5):1151. doi: 10.3390/ijms20051151 (PMC6429380; doi:10.3390/ijms20051151)

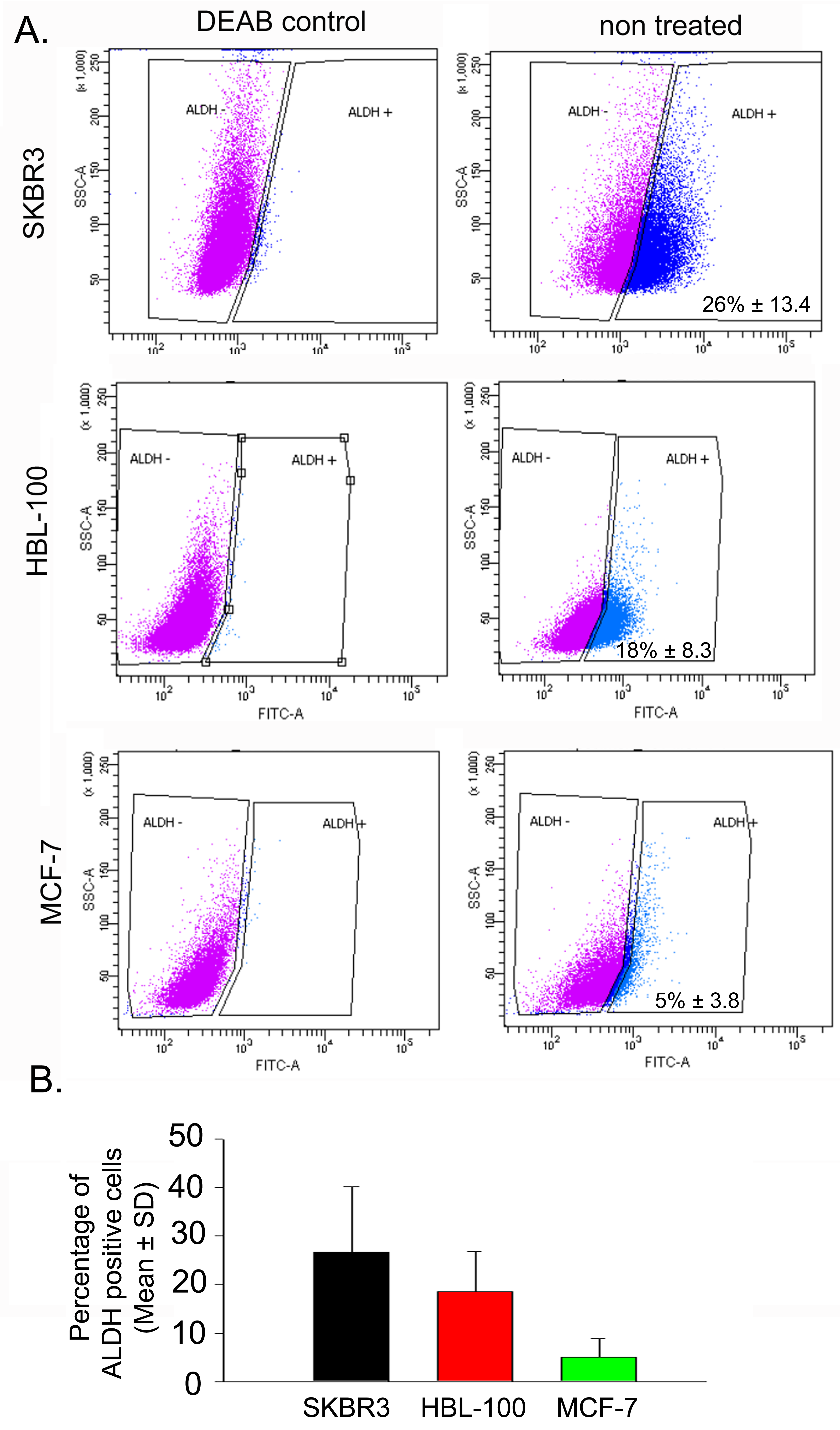

Supplement: Supplementary file 1 [file ijms-20-01151-s001.zip › Supplementary Figure S1.tif]

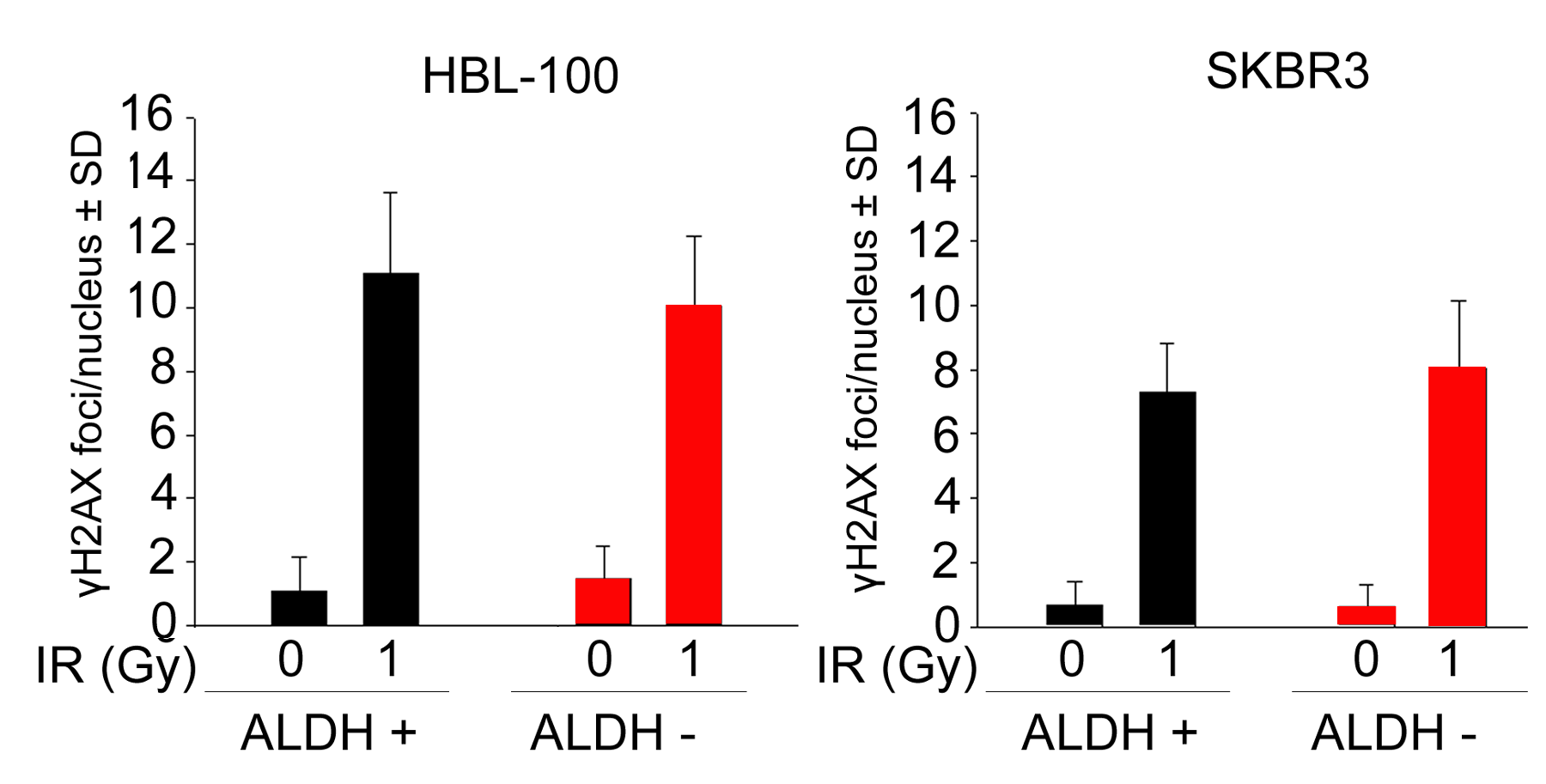

Supplement: Supplementary file 1 [file ijms-20-01151-s001.zip › Supplementary Figure S3.tif]

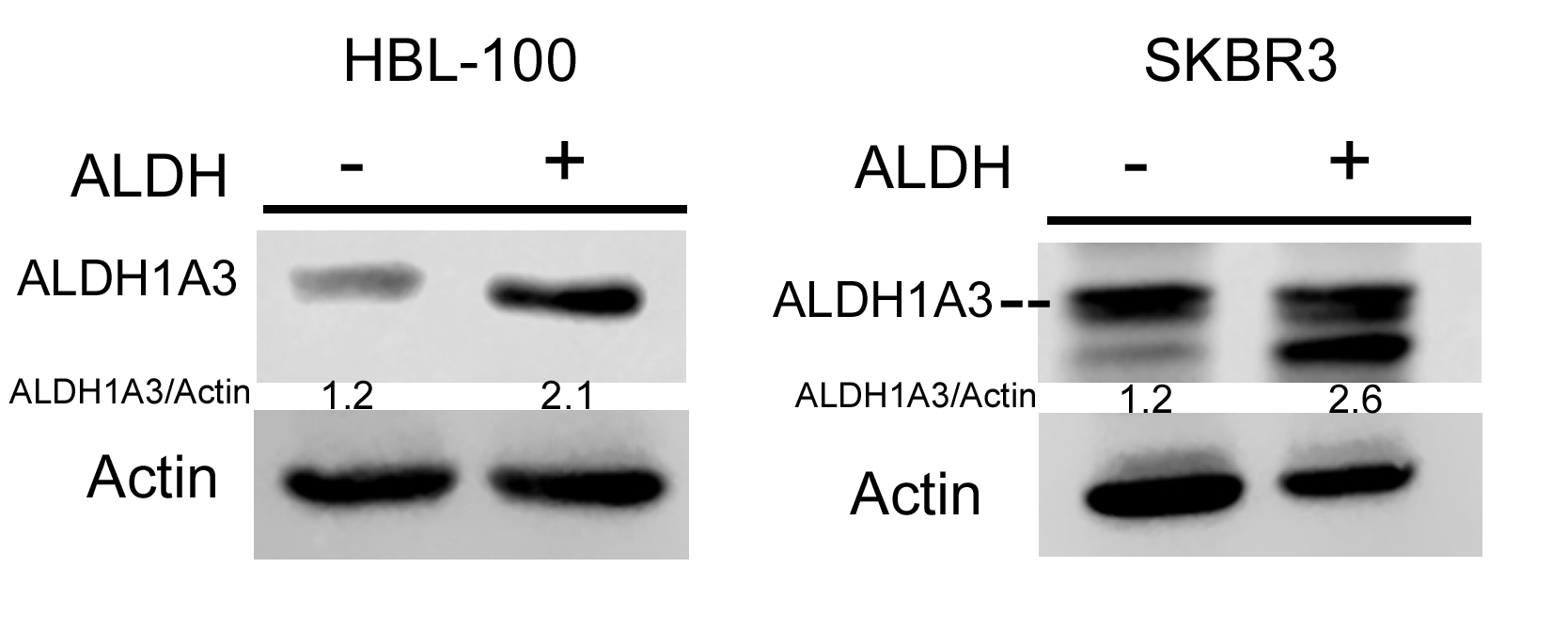

Supplement: Supplementary file 1 [file ijms-20-01151-s001.zip › Supplementary Figure S4.tif]

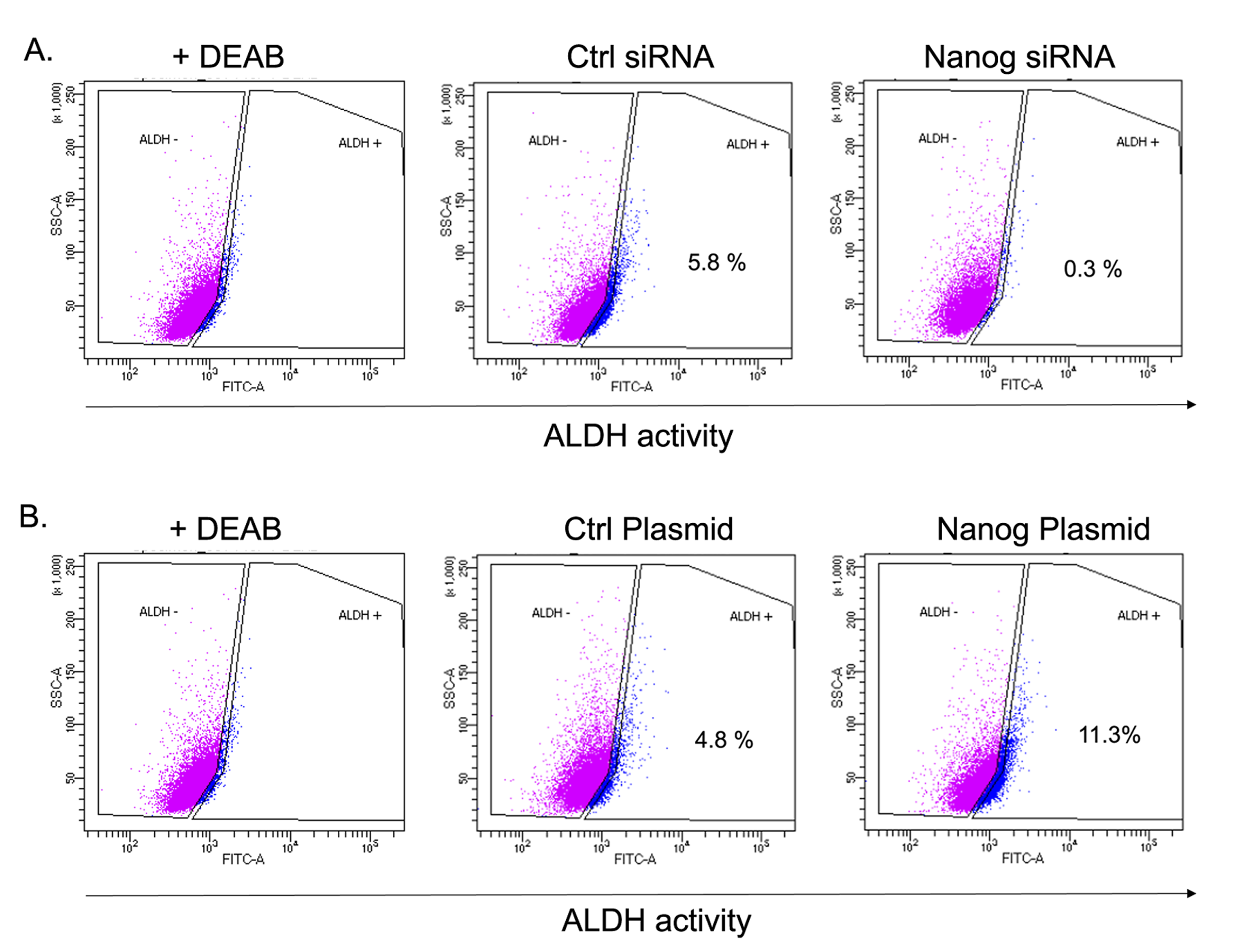

Supplement: Supplementary file 1 [file ijms-20-01151-s001.zip › Supplementary Figure S6.tif]

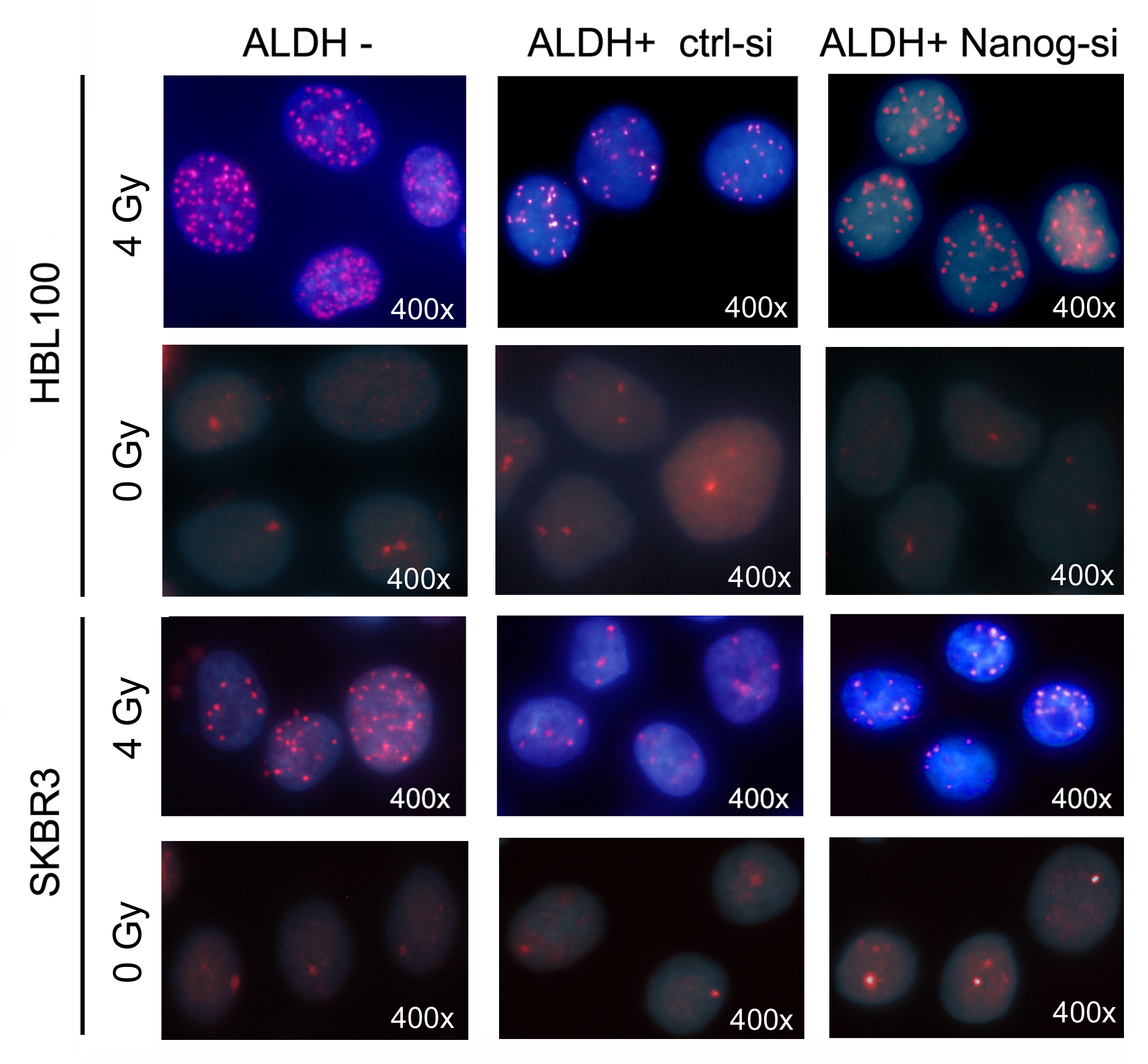

Supplement: Supplementary file 1 [file ijms-20-01151-s001.zip › Supplementary Figure S7.tif]

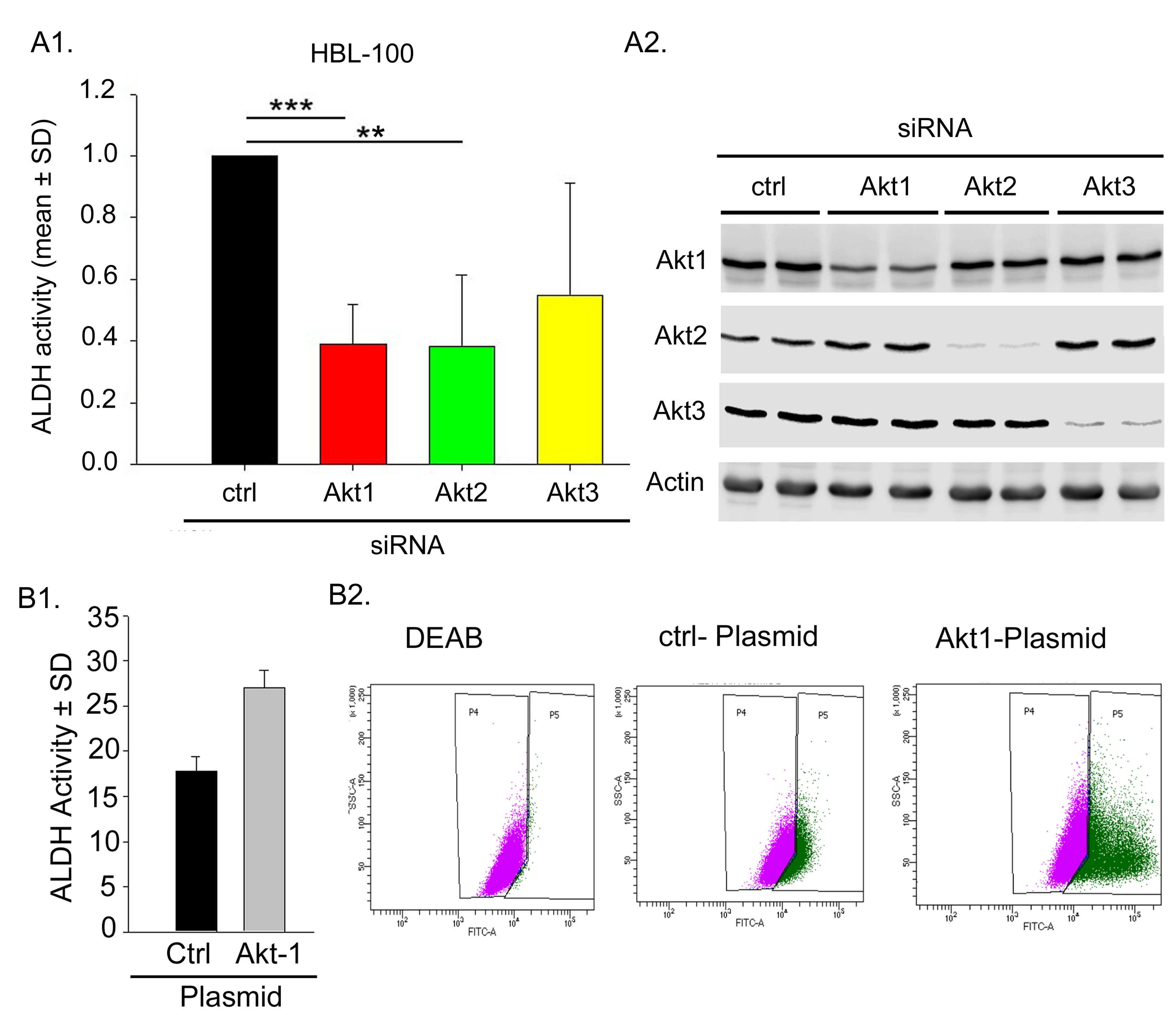

Supplement: Supplementary file 1 [file ijms-20-01151-s001.zip › Supplementary Figure S8.tif]

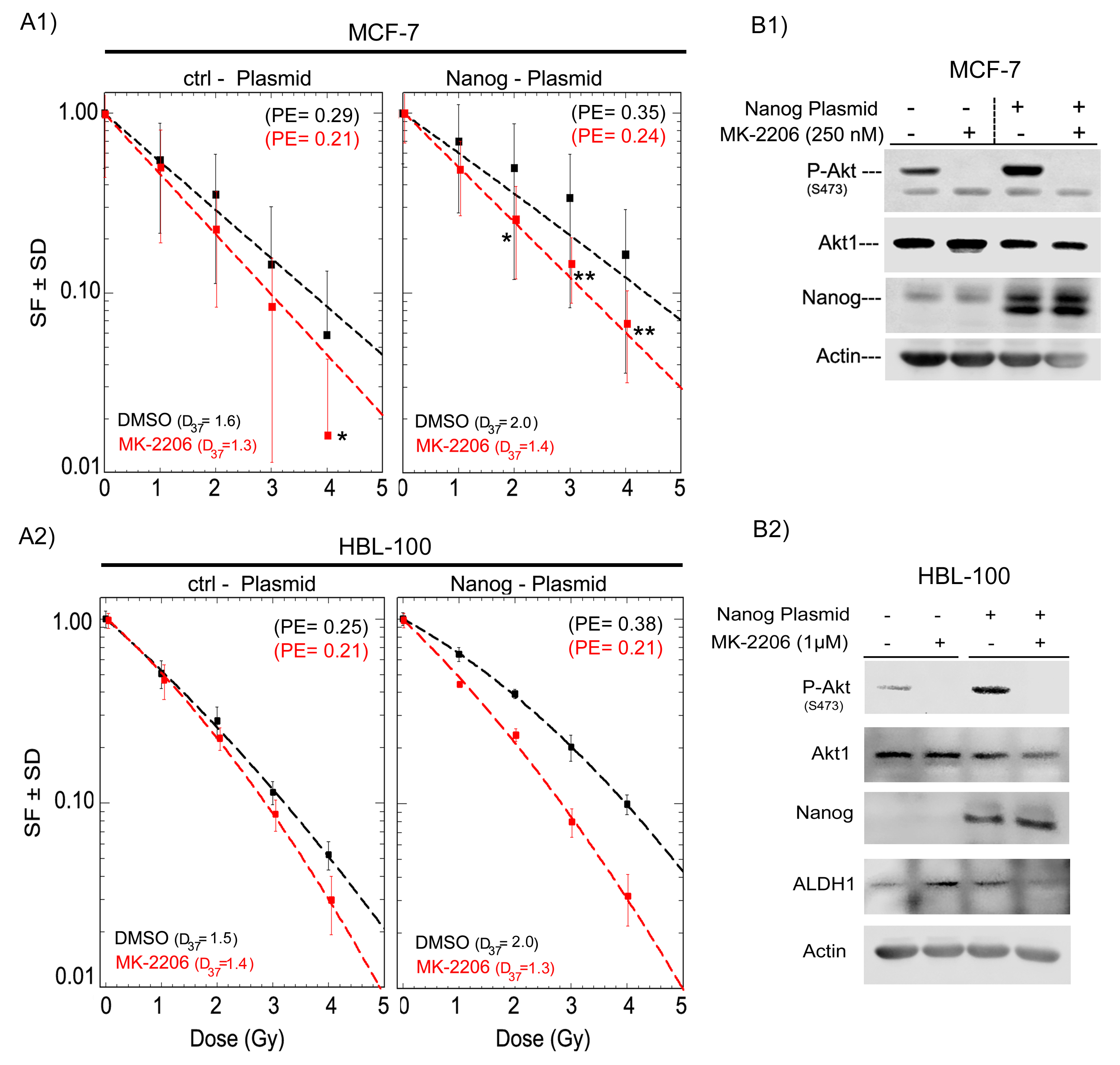

Supplement: Supplementary file 1 [file ijms-20-01151-s001.zip › Supplementary Figure S9.tif]

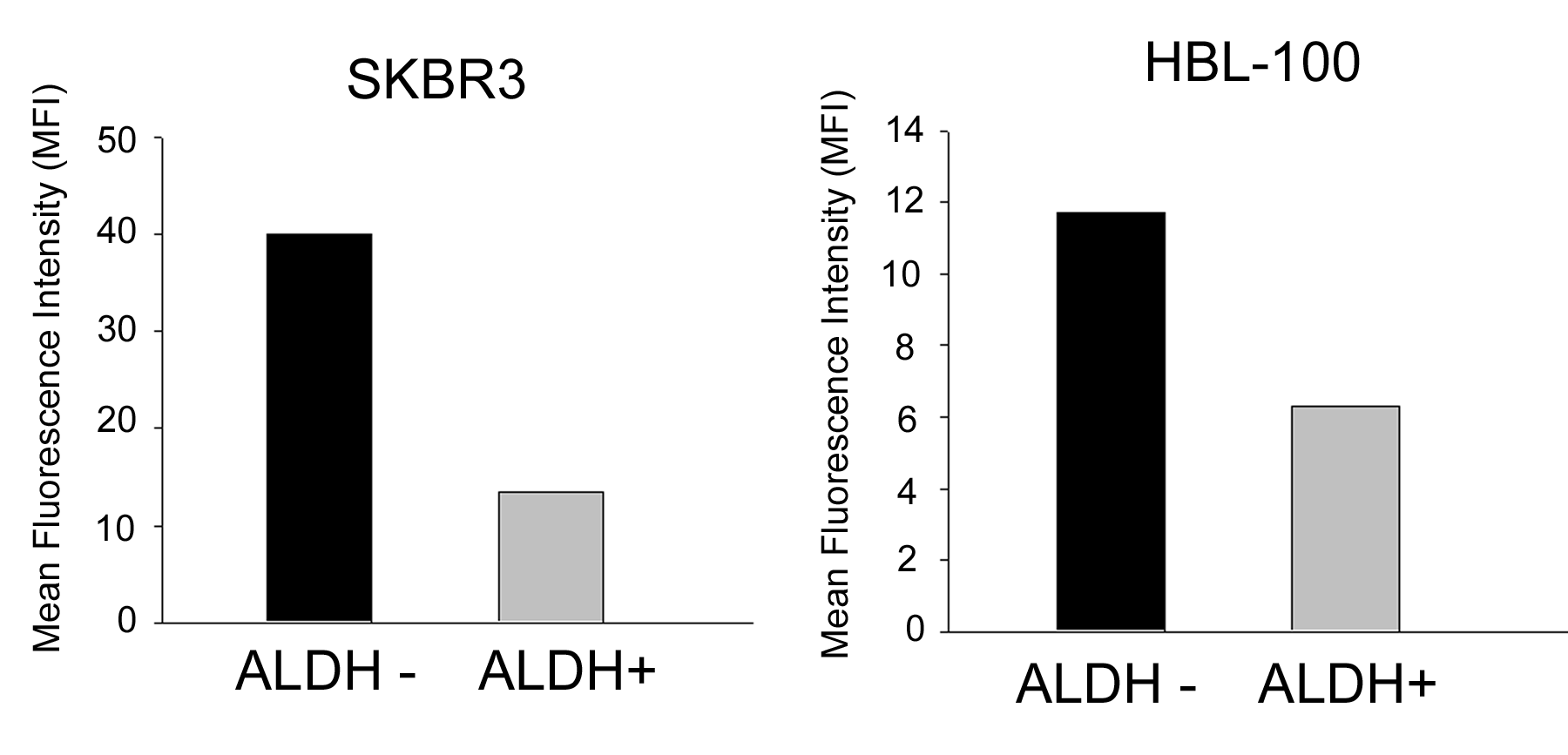

Supplement: Supplementary file 1 [file ijms-20-01151-s001.zip › Supplementray Figure S5.tif]
